# Points to Consider in Health Assessment of Adult Patients with Primary Antibody Deficiencies

**DOI:** 10.3390/jcm12186018

**Published:** 2023-09-17

**Authors:** Katarzyna Napiórkowska-Baran, Marcin Ziętkiewicz, Ewa Więsik-Szewczyk, Aleksandra Matyja-Bednarczyk, Marta Tykwińska, Ewa Alska, Tomasz Rosada, Ewa Szynkiewicz, Jakub Lubański, Oskar Schmidt, Bartłomiej Szymczak, Kinga Koperska, Zbigniew Bartuzi

**Affiliations:** 1 Department of Allergology, Clinical Immunology and Internal Diseases, Collegium Medicum Bydgoszcz, Nicolaus Copernicus University Torun, 85-067 Bydgoszcz, Poland; tykwinska@gmail.com (M.T.); e.alska@icloud.com (E.A.); tomaszrosada@poczta.onet.pl (T.R.); zbartuzi@cm.umk.pl (Z.B.); 2 Department of Rheumatology, Clinical Immunology, Geriatrics and Internal Medicine, Medical University of Gdansk, 80-210 Gdansk, Poland; zietmar@gumed.edu.pl; 3 Department of Internal Medicine, Pulmonology, Allergy and Clinical Immunology, Central Clinical Hospital of the Ministry of National Defense, Military Institute of Medicine, 04-141 Warsaw, Poland; ewa.w.szewczyk@gmail.com; 4 2nd Department of Internal Medicine, Jagiellonian University Medical College, 31-501 Krakow, Poland; matyjabed@gmail.com; 5 Department of Nursing in Internal Diseases, Collegium Medicum Bydgoszcz, Nicolaus Copernicus University Torun, 85-067 Bydgoszcz, Poland; ewaszynkiewicz@wp.pl; 6 Student Research Club of Clinical Immunology, Department of Allergology, Clinical Immunology and Internal Diseases, Collegium Medicum Bydgoszcz, Nicolaus Copernicus University Torun, 85-067 Bydgoszcz, Poland; kuba.albanski@gmail.com (J.L.); okischmidt@wp.pl (O.S.); bartlomiej.szymczak1@gmail.com (B.S.); kingakoperska@gmail.com (K.K.)

**Keywords:** inborn errors of immunity, primary immunodeficiencies, primary antibody deficiency, prevention, ferritin, folic acid, vitamin B12, vitamin D3, total protein

## Abstract

An improved recognition of inborn errors of immunity (IEI) is associated with an increase in life expectancy and a higher incidence of complications and related conditions. The aim of the study was to analyze factors enabling the primary prevention: BMI, smoking and selected laboratory tests (morphology with smear, creatinine, eGFR, total protein, albumin, ferritin, folic acid, vitamin B12, vitamin D3) included in the protocols of standard of care for adult patients with primary antibody deficiencies (PADs). The study included 94 participants ≥ 18 years old, diagnosed with PADs. Overweight was found in 17%, obesity in 14% and underweight in 15% of patients; 11.5% of patients smoked. Leukopenia was diagnosed in 16%, neutropenia in 8.5%, lymphopenia in 22.5% and thrombocytopenia in 14% of patients. A decreased concentration of hemoglobin was found in 32%, total protein in 19%, albumin in 17%, vitamin D3 in 52%, vitamin B12 in 6.5%, folic acid in 34% and ferritin in 26% of patients. Creatinine concentrations were elevated in 16% of patients, while in 20%, eGFR was reduced. Only a holistic assessment of comorbidities and complications of deficiency, as well as regular follow-up and lifestyle changes, can yield the best results in the long-term care of patients.

## 1. Introduction

The care of patients with primary immunodeficiencies (PIDs) is a challenge, both for family physicians and specialists, including clinical immunologists. Patients with inborn errors of immunity (IEI) are most commonly found to have humoral immune deficiencies, which, according to some analyses, account for up to 78% of all IEI [1,2]. Patients have an increased susceptibility to infections, autoimmune diseases, allergies and cancer [3,4]. The recognizability of IEI has been steadily improving, and this includes both a reduction in the time from symptom onset to diagnosis, as well as the precise identification of the genetic defect [5,6]. This is associated with a longer life expectancy of patients, and thus with a higher incidence of complications and conditions associated with immunodeficiency, as well as an increased risk of developing lifestyle diseases. The prevention and early detection of complications and the appropriate treatment of complications and comorbidities have become crucial in the care of this group of patients. On the other hand, the use of stimulants is a factor that has a negative effect on the abovementioned diseases and should be eliminated. Improvements in these aspects have become possible with the development and implementation of standard of care protocols for adult patients with primary antibody deficiencies (PADs) [7].

The aim of the study was to analyze factors that enable the introduction of primary prevention in adult patients with primary antibody deficiencies, such as: BMI, the use of addictive substances and selected laboratory tests (complete blood count with smear, creatinine, eGFR, total protein, albumin, ferritin, folic acid, vitamin B12, vitamin D3).

## 2. Materials and Methods

The study included an analysis of BMI, use of addictive substances and selected laboratory tests (CBC with smear, creatinine, eGFR, total protein, albumin, ferritin, folic acid, vitamin B12, vitamin D3), which were included in the author’s standard of care protocol for adult patients with PADs [7]. The study was conducted from June 2021 to October 2022. Inclusion criteria were age ≥18 years, diagnosis of IEI based on the criteria of the European Society for Immunodeficiencies (ESID) [8] and obtaining informed consent to participate in the study. The analysis was performed in 94 patients treated at the immunology center in Bydgoszcz, Poland. All participants were indigenous inhabitants of Central Europe (Caucasian race). The exclusion criteria were failure to meet the eligibility criteria and lack of consent to participate in the study. Blood for testing was collected during routine monitoring visits. The study was approved by the Bioethics Committee (KB 215/2022). Results are presented as arithmetic mean and standard deviation. Statistical analysis of correlations was performed using Pearson’s test. Statistical significance of the results was calculated utilizing t-statistic calculated from the coefficient value. The *p*-values were considered statistically significant when *p* was lower than 0.05. Calculations were performed using MS Excel 2019 with Analysis Toolpak add-in.

## 3. Results

### 3.1. Patients

In the study group consisting of 94 patients, the average age of patients, at the time of the study, was 41.3 ± 14.7 years. Women accounted for 57.5% (54 participants). The proportion of individual immunodeficiencies was as follows: common variable immunodeficiency (CVID), 30 patients (32%); unspecified hypogammaglobulinemia, 26 patients (28%); selective IgA deficiency, 12 patients (13%); deficiency of IgG subclasses, 12 patients (13%); selective IgM deficiency, four patients (4%); agammaglobulinemia, eight people (X-linked—four people (4%); autosomal recessive—four people (4%)); and immunodeficiency associated with other specified major defects, two participants (2%). Sixty-three participants (67%) received immunoglobulin supplementation, either subcutaneously (62 people—98.5%) or intravenously (one person—1.5%). The average time of remaining under the care of the immunology center in Bydgoszcz was 3.75 ± 3.31 years. The average delay in establishing a diagnosis of deficiency was 12.69 ± 13.94 years, noting that almost 70% of IEI were diagnosed in the last decade. Between 2018 and 2022, 39.5% of participants (37 patients) were diagnosed with IEI, while between 2013 and 2017, 30% (28 people) were diagnosed. The remaining 30.5% (29 patients) were diagnosed in 1985–2016. A detailed analysis of the patients is presented in Table 1 and Supplementary Materials.

### 3.2. Use of Addictive Substances

At the time of the study, 10 participants (10.5%) were smokers (nine smoked regularly, one occasionally). Five people (5.5%) declared a history of nicotinism. Two people (2%) abused alcohol. None of the patients used narcotics or other psychoactive substances and did not abuse medicines, e.g., analgesics or benzodiazepines.

### 3.3. Nutritional Status

Fifty patients (53%) were of normal weight, 17 people (18%) were overweight, 13 (14%) were obese and 14 participants (15%) were underweight.

### 3.4. Laboratory Tests

Fifteen patients (16%) had leukopenia, eight (8.5%) had neutropenia and 21 (22.5%) had lymphopenia. Thirty participants (32%) had decreased hemoglobin levels, with 63% of the cases being male. Thrombocytopenia was found in 13 patients (14%). Elevated creatinine levels were found in 15 participants (16%), while reduced eGFR was found in 19 patients (20%). Decreased total protein levels were found in 18 patients (19%), while decreased albumin levels in 16 patients (17%), while the total protein concentration was characterized by a statistically significant positive correlation with immunoglobulin supplementation.

Forty-nine participants (52%) were found to be vitamin D3 deficient, despite supplementation being recommended to all of the patients. Folic acid deficiency was diagnosed in 32 patients (34%), while vitamin B12 deficiency was found in only six participants (6.5%); however, five patients had a good response to oral supplementation and one patient suffered from pernicious anemia. Hypoferritinemia was detected in almost 30% of patients.

It should be emphasized that leukopenia, lymphopenia, neutropenia, trombocytopenia, anemia (both women and men), decreased concentrations of ferritin, total protein, albumin, vitamin D, vitamin B12 and folic acid were most common in patients with normal weight.

A detailed analysis of the laboratory results is shown in Table 2, Table 3 and Table 4 and in Supplementary Materials.

## 4. Discussion

The recognizability of IEI has been systematically improving. In the last decade, almost 70% of analyzed IEI cases have been diagnosed. Early detection of the deficiency is forcing a change in the approach to the care of patients with IEI, especially in terms of primary prophylaxis.

The center, under the care of which patients qualified for the study remain, is the youngest immunology center in Poland. Therefore, the average time of remaining under the center’s care was 3.75 ± 3.31 years. It is also one of the three centers with the largest number of IEI patients under its supervision. In order to provide patients with the best possible care, standards of care have been developed and implemented for PAD patients, who constitute the largest group of patients with IEI.

In 1974, Marc Lalonde introduced the concept of health fields, according to which modifiable factors such as lifestyle (50%) and environment (20%) have the greatest impact on human health of about 70%. Genetic factors and the organization of health care have a lower impact of 20% and 10%, respectively [9]. Therefore, special emphasis in patient care should be placed on lifestyle, which includes diet, physical activity, stress management skills and the use of addictive substances. An analysis of the available literature has shown that there are only a few studies which were available to introduce prophylaxis in patients with primary immunodeficiencies.

The complexity of the interaction between nutrition and the immune system is enormous. The functioning of this system is influenced by the overall nutritional status and the type of food intake (foods, nutrients, non-nutritive bioactive compounds). Conversely, the immune system influences metabolism, nutritional needs and physiological response to food [10]. Nowadays, nutrigenomics and immunonutrition (immunomodulatory nutrition) are gaining popularity, with the goal of improving or maintaining a patient’s nutritional status in preparation for, during or after treatment [11]. Oncologists, surgeons and gastroenterologists are already taking advantage of this knowledge [12,13].

The issue of obesity in PAD patients has been so far addressed in terms of immunoglobulin dosing or COVID-19 course [14,15,16]. Ruffner et al. emphasized that the prevalence of underweight and obesity in primary immunodeficiencies is unknown, despite the described associations between this group of conditions and weight loss or developmental disorders. The study included a retrospective analysis of 653 pediatric patients (2 to 20 years of age) and 514 adult patients (>20 years of age) using the United States Immunodeficiency Network (USIDNET). The prevalence of underweight and obesity in IEI patients was compared with data from the National Health and Nutrition Examination Survey (NHANES). In the pediatric group, patients were diagnosed not only with PAD, but also with, e.g., chronic granulomatous disease, severe combined immunodeficiency, Wiskott–Aldrich syndrome or complement deficiency. In the adult group, PADs dominated, but they were not the only deficiencies identified. Participants in the study were representatives of various nationalities (the current analysis included only Caucasians). Although the prevalence of obesity in PID patients was similar to that observed in the general population, it is important to emphasize the role of education about the impact of obesity on the course of the deficiency and chronic comorbidities in this group, with particular emphasis on cardiovascular risk. The authors also emphasized that both adults and children with PIDs had a significantly higher prevalence of underweight over multiple years of analysis. Further examination of underweight patients for PID diagnosis showed that underweight status in adults with CVID was associated with granulomatous disease, as well as with earlier age of CVID diagnosis. The analysis carried out by the authors of this study also showed that BMI weakly but statistically significantly (*p* < 0.05) correlated with the age of the first symptoms. Certainly, this small difference in the results is due to differences between the groups discussed above (Table S3 in Supplementary Materials) [17].

The use of addictive substances, including nicotine, should be covered in the care of patients with PADs. In a study by van der Poorten et al., a significant smoking history was declared by 38% of patients with CVID. In the analysis conducted by the authors of the present study, smoking was declared by 10.5% of patients, while 5.5% of patients declared a history of nicotinism. Arguably, this is related to patients’ greater awareness of smoking-related diseases, but still requires control and education [18].

Deficiencies of iron, folic acid, zinc, iodine, vitamin D and vitamin A are the most common micronutrient deficiencies worldwide [19,20], but not all measurements are widely available in daily practice. In the available literature, there are only single reports of vitamin D deficiencies in patients with humoral immunodeficiencies [21,22]. Research conducted by Cruz JRS et al. [21] and Amaya-Mejía AS et al. [22] included a very small group of subjects (15 and 20 patients with CVID, respectively). Vitamin D deficiency was found in 13% and 30% of patients, respectively. Vitamin D deficiency is a global problem. It has been estimated that approximately 30% and 60% of children and adults worldwide are vitamin D deficient and insufficient, respectively. Vitamin D deficiency is the cause of countless acute and chronic diseases, including periodontitis, autoimmune diseases, infectious diseases, cardiovascular diseases, fatal cancers, type 2 diabetes and neurological disorders [20]. Therefore, the measurement of the concentration of this parameter is particularly important in patients with immune system disorders. So far, there are no studies available in the literature on the assessment of ferritin and folic acid levels in the screening test in patients with PADs. There are also limited data on the prevalence of these deficiencies in the general population. In a study by Mézière A et al. on 14,904 samples taken from hospitalized patients regardless of the cause, folic acid deficiency was found in 20.4% and vitamin B12 deficiency in 4.6% of patients [23].

Folic acid plays a role in innate immunity (NK cells). It also plays a role in cellular immunity. It is essential for the proper response of antibodies to antigens and supports the immune response mediated by Th1. Vitamin B12 plays a role in the functioning of NK cells and is an immunomodulator of cellular immunity, especially with an effect on cytotoxic cells (NK cells, CD8+ T cells). It facilitates the production of T lymphocytes and is involved in humoral and cellular immunity. Iron, in turn, is involved in the regulation of the production and action of cytokines. It creates highly toxic hydroxyl radicals, taking part in the process of killing bacteria by neutrophils. It plays an important role in the differentiation and proliferation of T lymphocytes. It is necessary for cell differentiation and growth and is a component of enzymes crucial for the functioning of immune cells (e.g., ribonucleotide reductase involved in DNA synthesis) [24].

The ferritin/transferrin assay was used in the protocols because a single iron determination is of limited diagnostic value. Particularly in patients with IEI who often have inflammatory or autoimmune conditions. In addition, the obtained result does not answer the question of how long iron supplementation should be used. A low ferritin level is a good marker of iron deficiency. However, an elevated concentration is an additional clue to the existing inflammation. Of course, in doubtful cases, the concentration of ferritin should be correlated with the concentration of iron or other exponents of iron metabolism.

In the research conducted by the authors of this paper, folic acid deficiency were found in 34% and hypoferritinemia in almost 30% of patients. Information regarding these deficiencies is very important, as they further cause secondary immunodeficiency, which worsens the course of the underlying condition [25]. Although vitamin B12 deficiency was found in only 6.5%, it should be noted that this was characterized by a good response to oral supplementation. Data available in the literature confirm these observations and explain them, among other things, by changes in dietary habits, high food processing, or the abuse of certain medications (e.g., proton pump inhibitors) [26]. In the performed total protein and albumin test analysis, a reduction in total protein concentration was present in 19%, while albumin concentration was reduced in 17% of patients, which necessitated an additional verification of patients for the source of protein loss and malnutrition. In addition, albumin concentration allowed for the calculation of corrected calcium concentration. Moreover, abnormalities in the analyzed factors can affect the normal functioning of the bone marrow and thus the morphotic elements of the blood, causing, among other things, the aforementioned lymphopenia.

Blood count abnormalities in patients with IEI can be multifactorial. Patients have an increased risk of cytopenia, especially those of autoimmune origin [27], which may precede infectious complications [28]. Both cytopenia and elevated morphologic parameters can be a result of tumors, infections or medications. Therefore, patients with PADs should be regularly evaluated for these conditions.

As emphasized earlier, leukopenia, lymphopenia, neutropenia, thrombocytopenia, anemia (both female and male), decreased ferritin, total protein, albumin, vitamin D, vitamin B12 and folate levels were most common in patients with normal body weight. This is important information because nutrient deficiencies are often suspected in malnourished people. Unfortunately, due to the poor quality of food, these deficits also occur in people with a normal body weight.

The authors are aware that the research was conducted on a relatively small group of patients. However, the analysis made it possible to modify the recommendations in a significant number of patients. Multicenter studies covering a much larger group of patients are needed to perform accurate correlations and analyses. The authors hope that this paper will be an introduction to further research conducted not only in the group of patients with IEI, but also in comparison with the control group of healthy people.

## 5. Conclusions

An important point to emphasize is that all examined patients found the introduction of care protocols to be very desirable and helpful. All participants performed the recommended laboratory tests despite the ongoing COVID-19 pandemic, bearing in mind that unrecognized conditions could affect the course of not only the deficiency, but also SARS-CoV2 virus infection.

The performed analysis showed shortcomings in the primary and specialized care of IEI patients, and therefore, all physicians caring for IEI patients should have guidelines for multispecialty care at their disposal, including not only nutritional status and the use of addictive substances, but also certain laboratory tests.

The introduction of the protocol has improved the recognition of factors that may influence the course of immunodeficiency. It has confirmed that there is a need for careful analysis in establishing the diagnosis, in order to estimate complications and to plan follow-up diagnostic tests, especially with regard to nutritional status and selected laboratory tests. Moreover, it allowed for the introduction of optimal recommendations for patients with PADs, especially regarding diet, limiting the use of nephrotoxic drugs and the use of infection prophylaxis in selected patients with neutropenia and/or lymphopenia. Furthermore, it allowed the introduction of primary (primary) prophylaxis and thus possibly prolonging the life of patients and reducing the risk of disability.

Only a holistic assessment of comorbidities and complications of the deficiency, as well as regular check-ups and lifestyle changes, can yield the best results in the long-term care of patients with IEI.

## Figures and Tables

**Table 1:** General characteristics of patients.

		Result (N/100%)	
Parameter			
Sex		Female—54(57.5%)	Male—40 (42.5%)
Inborn Errors of Immunity		Common Variable Immunodeficiency (CVID) Unclassified Antibody Deficiency (UAD) Selective IgA Deficiency (SIgAD) IgG Subclass Deficiency (IgGSD) Selective IgM Deficiency (SIgMD) X-linked agammaglobulinemia (XRA) Autosomal Recessive Agammaglobulinemia (ARA) Immunodeficiency Associated With Other Specified Major Defects (IAODs)	30 (32%) 26 (28%) 12 (13%) 12 (13%) 4 (4%) 4 (4%) 4 (4%) 2 (2%)
Average Age (years)		41.3 ± 14.7	
Age at Diagnosis(years)		31.7 ± 18.5	
Age of Symptom Onset (years)		19.7 ± 17.1	
Average Delay in Diagnosis (years)		12.7 ± 13.9	
Immunoglobulin Replacement Therapy (IgRT)		Total Subcutaneous SCIG Intravenous IVIG Mean IgG concentration (mg/dL) Mean IgG dose (g/kg per month)	63 (67%) 62 (98.5%) 1 (1%) 803.98 ± 317.13 0.46 ± 0.27
Infections		Total Upper respiratory tract infections without sinusitis Sinusitis Lower respiratory tract infections Digestive tract infections Urinary tract infections Heart infections Skin infections Brain and meningeal infections	94 (100%) 86 (91.5%) 84 (89.5%) 49 (52%) 5 (5.5%) 13 (14%) 2 (2%) 7 (7.5%) 1 (1%)
Autoimmunity		Total Autoimmune Thyroid Disease (AITD) Inflammatory Bowel Disease (IBD) Autoimmune Hemolytic Anemia (AIHA) Immune Thrombocytopenia (ITP) Vasculitis (VAS) Rheumatoid Arthritis (RA) Psoriasis Psoriatic Arthritis (PA) Primary Adrenal Insufficiency (PAI) Addison–Biermer Anemia (ABA) Autoimmune Hepatitis (AH) Sjogren’s Syndrome (SS) Celiac Disease (CD) Graves’ Disease (GD) Myasthenia Gravis (MG) Antiphospholipid Syndrome (APS)	28 (30%) 7 (7.4%) *5 (5.5%)* 3 (3%) *3 (3%)* 3 (3%) 2 (2%) 2 (2%) 1 (1%) 1 (1%) 1 (1%) 1 (1%) 1 (1%) 1 (1%) *1 (1%)* 1 (1%) 1 (1%)
Body Mass Index (BMI)		Normal weight Overweight Obese Underweight	50 (53%) 17 (18%) 13 (14%) 14 (15%)
Smokers		Smoking currently Regularly Occasionally Smoked in the past	10 (10.5%) 9 (9.5%) 1 (1%) 5 (5.5%)
Alcohol Addiction		2 (2%)	

**Table 2:** Analysis of the laboratory results.

Parameter	Leukocytes (G/L)	Hemoglobin (g/dL)	PLT (G/L)	Neutrocytes (G/L)	Lymphocytes (G/L)	Creatinine (mg/dL)	eGFR (mL/min)	Vitamin D3 (ng/mL)	Folic acid (ng/mL)	Vitamin B12 (pg/mL)	Ferritin (ng/mL)	Total Protein (g/dL)	Albumin (g/dL)
** Reference ** **Values**	** 3.9–10.2 **	** F: 12–16 ** **M: 14–18**	** 130–400 **	** 1.5–7.7 **	** 1.1–4.4 **	** 0.51–0.95 **	** >90 **	** 30–50 **	** 4.6–18.7 **	** 191–663 **	** 30–400 **	** 6–8 **	** 3.5–5.2 **
Mean concentration	6.15 ± 2,37	F: 12.6 ± 1.17 M: 13.7 ± 1.76	231 ± 85	7.32 ± 1.93	1.67 ± 0.75	0.78 ± 0.24	105 ± 22	31.38 ± 15.3	6.95 ± 4.02	466.22 ± 236.42	127.32 ± 189.72	6.69 ± 0.76	3.98 ± 0.55
Values below reference	15 (16%)	30 (32%) F/M = 11/19	13 (14%)	8 (8.5%)	21 (22.5%)	6 (6.5%)	19 (20%)	49 (52%)	32 (34%)	6 (6.5%)	26 (27.5%)	18 (19%)	16 (17%)
Values above reference	8 (8.5%)	0(0%)	1 (1%)	5 (5.5%)	0 (0%)	15 (16%)	0%	10 (10.5%)	4 (4%)	18 (19%)	9 (9.5%)	1 (1%)	0 (0%)

**Table 3:** Pearson correlation coefficients between laboratory tests and clinical parameters of adult patients with primary antibody deficiencies (PADs). Statistically significant results (*p* < 0.05) are presented as bold text with the corresponding *p*-value. Underlined results suggest moderate or strong correlation. Positive results, i.e., greater than 0, indicate a positive correlation, while negative results, i.e., less than 0, represent a negative correlation.

Parameter	Leukocytes (G/L)	Hemoglobin (g/dL)	PLT (G/L)	Neutrocytes (G/L)	Lymphocytes (G/L)	Creatinine (mg/dL)	eGFR (mL/min)	Vitamin D3 (ng/mL)	Folic acid (ng/mL)	Vitamin B12 (pg/mL)	Ferritin (ng/mL)	Total protein (g/dL)	Albumin (g/dL)	Age (Years)	BMI	IgG Conc. (mg/dL)	CRP(mg/L)
Leukocytes (G/L)	1																
Hemoglobin (g/dL)	** 0.326 ** **(*p* = 0.0014)**	1															
PLT (G/L)	** 0.391 ** **(*p* < 0.0001)**	** 0.319 ** **(*p* = 0.0017)**	1														
Neutrocytes (G/L)	** 0.895 ** **(*p* < 0.0001 ) **	** 0.231 ** **(*p* = 0.0252)**	** 0.263 ** **(*p* = 0.0105)**	1													
Lymphocytes (G/L)	** 0.524 ** **(*p* < 0.0001 ) **	** 0.297 ** **(*p* = 0.0036)**	** 0.239 ** **(*p* = 0.0202)**	0.200	1												
Creatinine (mg/dL)	−0.054	0.021	** −0.226 ** **(*p* = 0.0284)**	−0.046	0.011	1											
eGFR (mL/min)	−0.008	** 0.254 ** **(*p* = 0.0133)**	0.169	−0.021	−0.025	** −0.744 ** **(*p* < 0.0001)**	1										
Vitamin D3 (ng/mL)	0.167	0.028	0.047	0.092	0.136	0.087	** −0.282 ** **(*p* = 0.0060)**	1									
Folic acid (ng/mL)	−0.140	0.017	−0.008	−0.105	−0.024	0.147	−0.118	0.198	1								
Vitamin B12 (pg/mL)	−0.028	0.090	0.139	−0.075	0.079	0.081	−0.038	0.136	** 0.289 ** **(*p* = 0.0048)**	1							
Ferritin (ng/mL)	0.046	−0.073	−0.014	0.119	−0.133	** 0.300 ** **(*p* = 0.0034)**	−0.130	−0.052	0.014	−0.031	1						
Total protein (g/dL)	0.046	** 0.486 ** **(*p* < 0.0001)**	** 0.380 ** **(*p* = 0.0002)**	−0.039	0.185	−0.082	0.103	0.053	0.055	−0.019	−0.151	1					
Albumin (g/dL)	−0.090	** 0.485 ** **(*p* < 0.0001)**	** 0.238 ** **(*p* = 0.0207)**	−0.182	0.153	−0.056	0.126	0.075	** 0.225 ** **(*p* = 0.0287)**	0.048	−0.096	** 0.804 ** **(*p* < 0.0001)**	1				
Age (years)	0.130	** −0.248 ** **(*p* = 0.0158)**	−0.022	** 0.206 ** **(*p* = 0.0465)**	−0.066	0.042	** −0.598 ** **(*p* < 0.0001)**	** 0.264 ** **(*p* = 0.0102)**	0.033	−0.058	0.098	−0.081	−0.125	1			
BMI	0.110	0.078	0.027	0.152	0.023	0.108	** −0.207 ** **(*p* = 0.0450)**	0.014	−0.061	−0.145	0.158	−0.022	0.037	** 0.258 ** **(*p* = 0.0121)**	1		
IgG Conc. (mg/dL)	−0.100	0.117	0.140	−0.113	0.018	0.110	−0.049	0.025	−0.078	−0.122	−0.005	** 0.485 ** **(*p* < 0.0001 ) **	** 0.232 ** **(*p* = 0.0243)**	−0.032	−0.110	1	
CRP (mg/L)	** 0.227 ** **(*p* = 0.028)**	** −0.239 ** **(*p* = 0.0203)**	0.010	** 0.342 ** **(*p* = 0.0008)**	−0.129	−0.161	0.096	−0.047	−0.123	0.027	−0.025	** −0.217 ** **(*p* = 0.0358)**	** −0.357 ** **(*p* = 0.0004)**	0.071	−0.044	−0.180	1

**Table 4:** Comparison of selected laboratory tests in relation to BMI.

	BMI (Weight Range)	Obese (BMI > 30)	Overweight (BMI 25–29.9)	Healthy Weight (BMI 18.5–24.9)	Underweight (BMI < 18.5)
Parameter:					
Amount of participants:		13 (14%)	17 (18%)	50 (53%)	14 (15%)
Leukopenia		1 (1%)	2 (2%)	7 (7.5%)	5 (5.5%)
Lymphopenia		3 (3%)	5 (5.5%)	9 (9.5%)	4 (4%)
Neutropenia		1 (1%)	1(1%)	4 (4%)	2 (2%)
Thrombocytopenia		0 (0%)	3 (3%)	6 (6.5%)	3 (3%)
Anemia		6 (6.5%)	8 (8.5%)	18 (19%)	6 (6.5%)
Females		2 (2%)	2 (2%)	4 (4%)	1 (1%)
Males		4 (4%)	6 (6.5%)	14 (15%)	5 (5.5%)
Vitamin D3 deficiency		5 (5.5%)	11 (11.5%)	27 (29%)	6 (6.5%)
Vitamin B12 deficiency		2 (2%)	1 (1%)	3 (3%)	0 (0%)
Folic acid deficiency		5 (5.5%)	7 (7.5%)	15 (16%)	5 (5.5%)
Ferritin		3 (3%)	2 (2%)	18 (19%)	3 (3%)
Albumin		1 (1%)	3 (3%)	7 (7.5%)	5 (5.5%)
Total protein		4 (4%)	3 (3%)	6 (6.5%)	5 (5.5%)

## Data Availability

The data presented in this study are available in supplementary material. Other data are available on request from the corresponding author. Some data is not publicly available due to the Personal Data Protection Regulation.

## Supplementary Materials

The following supporting information can be downloaded at: https://www.mdpi.com/article/10.3390/jcm12186018/s1, Table S1: General characteristics of patients. Table S2: Detailed analysis of laboratory tests. Table S3. Correlations of age of first symptoms, diagnosis and BMI.
