# Supplementary material for: Points to Consider in Health Assessment of Adult Patients with Primary Antibody Deficiencies
Source: J Clin Med. 2023 Sep 17;12(18):6018. doi: 10.3390/jcm12186018 (PMC10532356; doi:10.3390/jcm12186018)
Supplement: Supplementary file 1 [file jcm-12-06018-s001.zip › jcm-2467435-supplementary.pdf]

Journal of
Clinical Medicine

Table S1. General characteristics of patients

Parameter / Patient number	Age (years)	Sex	IEI	Age at diagnosis (years)	Age of symptom onset (years)	IgRT	IgRT dose (g/kg per month)	IgG Concentration (mg/dl)	Infections	Autoimmunity	BMI
1	67	F	IgGSD	62	54	-	-	738	2,3	-	29
2	63	F	UAD	57	51	IVIG	0,39g	628*	1,2,3,5	RA	29.7
3	20	F	IAOD	13	1	SCIG	0,43	645*	1,2,3	-	27.3
4	32	M	CVID	2	2	SCIG	0,38	711*	1,2,3	-	24.1
5	35	M	UAD	28	24	SCIG	0,58	1040*	1	-	16.3
6	28	F	CVID	16	1	SCIG	0,58	1270*	1,2,3	-	20.4
7	52	M	UAD	49	33	-	-	400	1	PA	23.6
8	78	M	UAD	72	55	SCIG	0,42	1070*	4,5	-	24.5
9	21	M	IgGSD	2	1	SCIG	0,40	1280*	1,2,3	IBD	30.1
10	40	M	IgGSD	34	14	SCIG	0,41	911*	1,2,7	ITP	21.8
11	24	M	XLA	1	1	SCIG	0,47	849*	1,2,3,7	-	19.66
12	40	M	SIgAD	37	4	-	-	1660	1,2	PAI	15.8
13	25	F	SIgAD	7	6	-	-	704	1,3	-	14.22
14	27	F	IgGSD	9	5	-	-	1380	1,4	-	18.4
15	36	M	CVID	27	24	SCIG	0,27	454*	1,2,3,	-	39.0
16	26	M	SIgAD	22	20	-	-	1550	1,2	-	19.8
17	40	F	CVID	35	30	SCIG	0,53	1450*	1,2	-	19.6
18	38	M	CVID	31	24	SCIG	0,35	1100*	1,2,3,7	AIHA	26.2
19	36	F	SIgAD	34	24	-	-	916	3	AITD, ABA	23.4
20	57	M	CVID	50	12	SCIG	0,45	417*	1,2,3	-	22.1
21	36	F	IgGSD	35	34	-	-	903	1,2,3	AIDT	22.5
22	43	F	UAD	38	34	-	-	1190	1,2	AITD	19.26
23	41	F	CVID	37	13	SCIG	0,45	190*	1,2,5	AIHA, ITP	25.78
24	29	F	ARA	25	1	SCIG	0,6	656*	1,2,5	-	15.6
25	23	M	CVID	12	2	SCIG	0,5	787*	1,2,3	-	20.0
26	27	F	CVID	13	10	SCIG	0,47	706*	1,2,3	-	22.1
27	56	M	UAD	52	40	SCIG	0,5	989*	1,2	-	19.5
28	50	F	SIgAD	44	13	-	-	1760	1,2	AITD	21.5
29	41	M	CVID	35	35	SCIG	0,3	870*	1,2,6	Psoriasis	30.6
30	43	F	UAD	41	19	-	-	543	1,2,3,6	-	20.4
31	22	M	IAOD	4	1	SCIG	0,68	1380*	1,2,3	-	16.6
32	58	F	ARA	56	48	SCIG	0,53	528*	1,2,3	-	19.7
33	48	F	UAD	43	23	-	-	612	1,2	-	22.3
34	40	F	CVID	28	25	SCIG	0,50	78*	1,2,3	-	20.8
35	61	F	UAD	-	-	-	-	429	1,2	-	33.12
36	38	F	CVID	36	1	SCIG	0,59	824*	1,2,3	-	17.0
37	61	F	UAD	59	20	-	-	578	1,2,3	-	20.1
38	36	F	UAD	34	1	SCIG	0,58	827*	1,2	AH, AITD	19.0
39	48	F	CVID	44	39	SCIG	0,47	1000*	1,2	-	21.9
40	19	M	UAD	9	1	SCIG	0,59	807*	1,2,4	IBD	17.4
41	58	M	CVID	49	46	SCIG	0,46	999*	1,2,3,5	-	22.49
42	51	F	UAD	48	7	-	-	609	1,2,5	-	25.3
43	27	F	IgGSD	3	2	-	-	748	1,2,5	-	27
44	38	M	SIgMD	32	30	-	-	1400	1,2	-	19.26
45	37	F	SIgAD	35	32	-	-	1860	1	AITD	21.51
46	67	M	UAD	55	64	SCIG	0,29	751*	1,2	VAS	30.1

47	21	M	IgGSD	14	1	SCIG	0,31	919*	1,2	-	29.8
48	39	M	CVID	28	26	SCIG	0,5	510*	2	IBD	19.38
49	63	F	IgGSD	59	14	SCIG	0,40	1140*	1,2,3	SS	31.2
50	19	F	UAD	7	7	SCIG	0,36	719*	1,2	-	20.0
51	33	F	CVID	17	1	SCIG	0,47	551*	1,2,7	-	27.5
52	23	F	SIgMD	17	13	-	-	867	1	-	24.6
53	21	M	ARA	9	8	SCIG	0,46	935*	1,2,8	-	23.7
54	32	F	IgGSD	28	15	-	-	999	1,2	-	21.2
55	20	M	XLA	6	6	SCIG	0,37	871*	2,3	-	18.8
56	28	M	CVID	13	8	SCIG	0,31	570*	1,2	-	20.2
57	48	F	CVID	35	32	SCIG	0,58	887*	1,2,3,4	-	18.9
58	65	F	IgGSD	59	4	SCIG	0,47	1060*	1,2,3,5	-	26.2
59	46	F	SIgAD	42	23	-	-	2170	1,2,3,5	-	19.7
60	39	F	ARA	36	4	SCIG	0,44	336*	1,2,3	-	22.00
61	41	F	UAD	31	14	SCIG	0,39	933*	1,2,5	-	21.6
62	34	F	IgGSD	12	1	SCIG	0,5	933*	1,2,3,5	-	22.66
63	42	M	XLA	4	4	SCIG	0,67	394*	1,2,3,7	-	18.1
64	47	F	CVID	41	31	SCIG	0,58	1120*	1,2,3	-	24.45
65	27	F	IgGSD	16	9	SCIG	0,43	956*	1,2,3	-	25.7
66	29	F	UAD	4	4	-	-	1720	1,2	-	16.18
67	42	M	SIgAD	38	36	-	-	1640	1,2	IBD	25.06
68	20	M	SIgMD	7	18	-	-	1170	1,2	AITD	21.6
69	54	F	UAD	53	5	SCIG	0,47	780*	1,2,3	-	23.8
70	44	F	CVID	19	10	SCIG	0,41	700*	1,2,3,4	CD, IBD	29.26
71	20	F	SIgAD	10	9	-	-	930	1	-	20.32
72	50	M	CVID	47	23	SCIG	0,42	983*	1,2,3	VAS, psoriasis	25.5
73	65	F	SIgAD	63	47	-	-	1530	1,2,3,5		41.02
74	50	F	UAD	46	46	SCIG	0,56	1150*	1,3,3	-	19.9
75	37	M	CVID	31	29	SCIG	0,35	868*	1,2,3	AIHA	25.9
76	28	M	CVID	27	27	SCIG	0,39	23,1*	1,2	-	23.8
77	49	F	CVID	27	1	SCIG	0,32	490*	1,2,3	-	34.6
78	30	F	CVID	15	7	SCIG	0,58	1160*	1,2,3	-	17.2
79	39	F	CVID	37	29	SCIG	0,5	407*	1,2,3	-	22.3
80	29	M	UAD	6	1	SCIG	0,32	888*	1,2	-	31.4
81	65	F	CVID	52	32	SCIG	0,74	1300*	1,2,3	-	15.99
82	55	F	UAD	49	47	SCIG	0,33	953*	1,2,3	VAS	31.89
83	56	F	UAD	48	48	-	-	509	1,2,7	-	41.4
84	62	F	SIgMD	55	55	-	-	903	1,2,3,5	GD	29.41
85	40	F	UAD	31	29	SCIG	0,47	1170*	1,2,3	-	24.6
86	41	M	CVID	23	15	SCIG	0,47	435*	1,2,3	-	23.2
87	58	M	SIgAD	52	46	-	-	1400	2,3	-	38.31
88	24	M	SIgAD	17	1	-	-	1440	1,2	-	30.9
89	41	F	UAD	40	6	SCIG	0,66	431*	1,2,3	MG	15.8
90	73	F	UAD	67	4	-	-	465	7	-	19.5
91	64	F	UAD	59	58	SCIG	0,68	1160*	1,2,3	RA	16.4
92	41	M	CVID	36	32	SCIG	0,35	794*	2,3	APS, ITP	25.7
93	69	M	CVID	58	20	SCIG	0,6	1240*	1,2,3	-	21.1
94	35	M	XLA	3	1	SCIG	0,35	936*	1,2,3	-	24.6

Abbreviations: **F** - Female, **M**– Male**, IEI** –Inborn Errors of Immunity, 1 - Upper respiratory tract infections without sinusitis, 2 - Sinusitis, 3 - Lower respiratory tract infections, 4 - Digestive tract infections 5 - Urinary tract infections, 6 - Heart infections, 7- Skin infections, **8** - Brain and meningeal infections, **ARA** – Autosomal Recessive Agammaglobulinemia, **CVID** – Common Variable Immunodeficiency, **IAOD** – Immunodeficiency Associated With Other Specified Major Defects, **IgGSD** – IgG Subclass Deficiency, **SIgAD** – Selective IgA Deficiency, **SIgMD** – Selective IgM Deficiency, **XLA** - X-linked agammaglobulinemia, **UAD** – Unclassified antibody deficiency, **IgRT** - Immunoglobulin Replacement Therapy, **IVIG** – Intravenous IgRT, **SCIG** – Subcutaneous IgRT, **RA** -

Rheumatoid Arthritis, **PA** - Psoriatic Arthritis, **IBD** - Inflammatory Bowel Disease, **ITP** - idiopathic thrombocytopenic purpura, PAI - Primary Adrenal Insufficiency, **AIHA** - Autoimmune Hemolytic Anemia, **AITD** - Autoimmune Thyroid Disease, **ABA** - Addison–Biermer Anemia, **AH** - Autoimmune Hepatitis, **VAS** - vasculitis, **SS** - Sjogren's syndrome, **CD** - celiac disease, **GD-** Graves' disease**, MG** - Myasthenia Gravis, **APS** - Antiphospholipid Syndrome,, * IgRT, **BMI -** Body Mass Index

Table S2. Detailed analysis of laboratory tests.

**Parameter**	**WBC (G/l)**	**HGB (g/dl)**	**PLT (G/l)**	**Neutro-cytes (G/l)**	**Lympho-cytes (G/l)**	**Creatinine (mg/dl)**	**eGFR (ml/min)**	**Vitamin D3 (ng/ml)**	**Folic acid (ng/ml)**	**Vita-minB12 (pg/ml)**	**Ferritin (ng/ml)**	**Total protein (g/dl)**	**Albumin (g/dl)**	**CRP (mg/l)**
**Reference values / Patient number**	**3,9-10,2**	**F:12-16 M:14-18**	**130-400**	**1,5-7,7**	**1,1-4,4**	**0,51-0,95**	**>90**	**30-50**	**4,6-18,7**	**191-663**	**30-400**	**6-8**	**3,5-5,2**	**<5**
1	4,77	13,7	276	2,56	1,46	0,8	78	28,51	13,7	382,7	98	6,76	4,06	<0,6
2	7,35	12,1	243	4,18	2,29	0,55	102	13,18	4,2	438,7	30	6,69	3,69	12
3	6,12	12,4	133	2,39	3,11	0,47	145	44,99	2,8	593,6	148	6,02	3,52	5,8
4	6,77	13,7	243	4,26	1,54	0,89	115	18,78	10,7	329,7	11	6,43	3,29	9,1
5	2,18	10,6	48	1,66	0,27	2,05	41	5,31	16,1	776,3	908	4,8	2,98	0,7
6	2,99	13,2	252	1,3	1,25	0,6	126	23,63	5,8	292,1	17	6,64	4,23	<0,6
7	6,26	15,6	221	3,29	2,19	0,91	98	43,46	18,07	474	28	6,5	5,05	<0,6
8	7,35	13,4	398	5,0	1,63	1,03	70	53,2	8,8	253,1	496	6,49	3,73	1,1
9	7,82	15,4	228	3,38	3,05	0,9	123	43,1	2,4	337,3	131	7,59	4,39	1,5
10	5,51	16,6	286	2,19	1,63	0,75	116	23,74	10,0	348,3	183	7,29	4,46	5,4
11	6,62	17,4	196	4,24	1,77	0,8	127	34,97	2,4	487	74	5,94	2,67	1,1
12	6,62	17,4	196	4,24	1,77	0,9	108	61,55	11,3	647,4	74	7,98	4,81	<0,6
13	9,61	13,2	367	6,14	1,46	0,53	133	49,88	9,1	776,6	71	5,93	3,33	3,1
14	6,76	13,9	342	3,51	1,1	0,48	136	37,66	6,4	600,6	101	7,0	3,54	6,6
15	7,04	14,7	303	5,02	1,21	0,57	135	17,82	7,2	241,3	197	6,72	4,02	8,0

16	5,86	12	264	3,61	1,56	1,16	88	18,43	5,6	428,1	11	6,47	3,88	0,7
17	2,8	13,3	186	1,6	0,91	0,74	103	31,79	14,9	390,0	12	7,26	4,38	0,8
18	6,15	12	161	5,03	0,72	0,7	122	22,3	4	510	623	5,85	3,66	2,1
19	3,18	11,3	64	1,08	1,39	0,53	124	14,13	8,3	76,04	171	6,01	4,06	1,3
20	5,21	13,9	336	2,86	1,25	0,47	125	24,19	1,9	218,5	215	6,8	3,59	<0,6
21	9,01	12,5	264	2,62	1,45	0,73	107	44,28	3,3	353,2	7	6,54	3,84	1,0
22	4,23	11,3	305	2,49	1,38	0,75	98	29,11	8,0	593,1	2	7,8	4,6	<0.6
23	3,32	12,1	110	1,86	0,91	1,59	38	77,27	>40	1061	136	6,46	4,12	4,1
24	8,59	13,2	236	4,75	3,15	0,85	94	49,3	4,4	347,3	37	7,32	4,27	1,7
25	6,18	12,4	233	4,26	1,02	0,79	129	8,78	3,3	370	573	6,44	3,72	30,6
26	6,61	12,9	348	4,0	2,01	0,64	124	40,24	5,7	300,6	28	6,57	3,82	6,7
27	13,96	14,4	250	8,49	4,09	0,99	86	31,97	6,5	629,3	282	6,68	3,57	12,5
28	4,67	12,7	160	1,95	2,31	0,72	99	37,67	9,6	198,9	53	7,08	4,21	1,7
29	12,23	15,4	222	10,2	1,25	1,03	91	57,73	5,7	289,2	150	5,92	3,52	23,4
30	5,88	11,1	227	2,68	1,89	0,79	93	37,79	11,8	505,4	11	5,95	3,58	1,8
31	2,94	15,1	144	1,09	1,15	0,83	127	27,19	2,9	505,3	64	7,42	4,78	<0,6
32	11,04	13	246	8,04	2,4	0,5	109	27,8	9,9	552,8	115	5,89	3,7	25,4
33	3,42	12	153	2,24	0,83	0,46	120	53,24	11	1238	29	6,59	4,4	<0,6
34	2,95	10,3	85	1,82	0,75	0,91	81	22,79	7,7	438	6,37	6,76	4,08	1,1
35	6,59	14,2	251	4,71	1,23	0,55	102	35,87	5,4	767,9	33	7,08	4,49	2,4
36	7,65	12,1	326	4,46	2,02	0,79	97	14,38	3,8	1278	69	5,18	2,79	2,3
37	6,26	12,7	195	2,88	2,43	0,68	96	19,56	5,3	216,5	45	5,4	3,46	<0,6
38	4,15	12,6	356	2,18	1,31	0,55	123	14,72	8,6	253,6	7	7,72	4,76	1,6
39	7,59	12,6	300	4,11	2,57	0,88	80	27,65	2,7	418,6	8,7	6,51	3,93	<0,6
40	4,72	14,5	270	2,14	2,08	0,7	138	33,7	17,6	738,6	23	7,11	4,69	0.9
41	10,64	16,6	376	8,13	1,53	0,79	100	31,74	3,4	455,1	401	7,63	4,41	1,4

42	11,05	14,1	214	7,79	2,37	0,7	102	25,31	4,8	348,6	31	7,13	4,24	0,83
43	7,12	15,4	225	5,57	0,93	0,56	129	22,34	4,8	177,4	126	7,49	4,19	4,8
44	3,95	14,4	215	1,91	1,44	1,01	95	52,93	4,4	342,6	51	7,10	4,02	<0,6
45	5,2	11,9	257	3,03	1,15	0,56	121	23,72	5,5	327,2	12	7,64	4,29	10
46	7,85	13,8	165	6,74	0,68	0,97	81	43,33	8,9	182,2	50	5,9	3,99	2,1
47	9,39	15,1	213	4,99	3,23	0,98	112	9,2	7,5	217,4	123	6,6	4,16	<0,6
48	4,83	11,5	102	3,75	0,6	0,85	111	6,75	7,7	105,2	174	6,10	3,48	1,8
49	5,85	12,9	255	4,27	1,01	0,91	69	20,73	2,1	343	44	6,87	3,65	3,0
50	4,91	12,9	164	2,04	2,46	0,64	131	16,08	6	394,9	170	7,22	4,5	1,5
51	1,95	10,9	120	0,96	0,84	0,47	133	17,35	3,5	404,3	45	5,34	2,86	13,4
52	5,7	13,1	231	3,87	1,17	0,59	131	19,25	5,0	329,2	41	6,68	4,27	2,4
53	6,89	14,9	181	3,15	2,89	0,92	121	31,84	7,7	349,4	225	7,13	4,21	2,8
54	5,69	12,2	153	2,32	2,69	0,91	85	42,81	4,3	179,9	37,4	6,1	3,32	3,6
55	6,31	15,8	387	2,8	2,33	0,67	140	22,58	>40	780,6	74	7,6	4,98	0,7
56	6,39	15,8	184	3,29	2,63	0,68	134	10,5	2,8	723,6	41	7,05	4,56	<0,6
57	1,36	8,6	37	0,54	0,68	0,65	107	24	3,6	293,9	11	4,44	2,66	4,2
58	7,58	14,4	261	3,73	2,62	0,93	66	38,65	4,1	293,3	50	7,99	4,61	4,6
59	4,94	12,4	277	3,01	1,35	0,67	109	31,66	10,6	302,2	18	8,27	4,71	0,8
60	5,89	12	307	3,17	0,79	0,6	128	43,63	3,2	287,5	1102	6,27	4,31	1,4
61	6,39	13,1	125	3,87	1,64	1,21	75	26,85	4,9	474,7	19	7,64	4,54	1,2
62	5,9	13,7	364	3,59	0,86	0,55	125	24,56	6,0	406,6	48	6,88	3,78	1,0
63	7,7	10,4	71	7,12	0,33	0,63	123	27,67	3,2	219,2p	48	5,29	2,49	136,6
64	6,8	12,9	260	4,92	1,33	0,53	115	34,88	6,1	236,9	26	7,17	4,20	9,3
65	7,92	14	269	4,97	2,38	0,63	125	26,12	5,3	379,1	128	7,19	4,16	39,4
66	3,64	12,4	311	2,0	1,38	0,69	120	25,58	9,0	541,0	28	7,63	4,35	0,9
67	6,76	13,7	293	4,61	1,37	0,87	107	27,87	7,2	347	96	7,39	4,18	1,7

68	7,09	15,1	326	3,12	2,87	1,13	94	19,36	8,4	674,9	55	7,41	4,12	0,8
69	5,19	13	242	1,56	3,07	0,61	105	31,13	16,9	908,4	49	6,0	3,9	<0,6
70	4,68	12	242	2,05	2,09	0,63	111	57,84	6,4	969,1	22	6,55	3,93	34,6
71	6,11	14,8	206	3,56	2,1	0,8	108	22,31	10,3	335,3	20	7,38	4,83	<0,6
72	5,88	10,9	191	6,35	1,57	0,68	113	14,49	19,9	294,1	476	6,15	3,17	32,3
73	5,24	11,8	215	2,62	1,84	1,22	47	52,22	4,2	500,6	644	6,54	3,75	4,2
74	5,9	12,5	314	2,99	2,23	0,87	79	92,6	8,6	314,8	45	6,72	4,04	<0,6
75	6,05	13,8	69	4,76	0,84	0,92	107	26,62	3,7	410,5	56	5,77	3,28	3,7
76	8,32	15,2	258	4,9	2,33	0,93	112	45,25	4,9	410,3	56	6,17	4,40	1,7
77	6,74	13,1	157	4,79	1,33	0,68	105	36,6	10,8	515,4	24	5,8	3,74	9,4
78	3,15	13,7	205	0,79	1,57	0,72	114	33,02	4,5	384,3	2,25	6,47	3,91	1,1
79	10,53	11,5	466	6,9	1,85	0,63	115	27,8	4,1	810,6	63	5,89	3,19	130
80	1,37	16,1	232	0,3	0,95	0,98	105	10,08	9,1	831,1	212	7,8	4,77	5,9
81	5,92	13,2	171	4,25	0,93	0,55	100	26,14	6,8	501,8	95	7,6	4,29	61,2
82	4,63	12,6	184	2,56	1,11	0,69	100	31,91	2,8	260,7	8	6,01	3,82	21,9
83	4,96	10,5	255	2,42	1,97	1,26	48	18,33	3,0	121,8	111	5,77	3,38	7,2
84	7,19	13	231	5,08	1,51	0,59	99	52,32	>40,0	553,5	132	6,75	4,16	1,9
85	5,57	12,7	400	3,61	1,3	0,71	108	28,32	7,2	668,2	12	7,59	4,40	1,9
86	7,55	13,6	232	5,45	1,57	0,67	100	30,25	2,2	594	34	6,38	3,92	24,6
87	10,45	13,7	246	6,89	2,72	0,92	92	49,43	6,0	391,7	478	7,51	4,45	<0,6
88	6,67	15,8	342	4,29	1,8	0,83	125	7,46	9,6	407,9	253	7,91	4,97	1,1
89	3,23	10,7	113	1,79	1,07	0,62	113	35,31	17,1	301	59	5,64	3,68	<0,6
90	5,37	12,8	203	2,28	1,9	0,64	89	37,19	10,5	679,9	265	6,99	4,3	1,1
91	11,16	12,3	254	6,94	3,1	0,8	79	46,53	6,2	644,9	46	7,03	3,28	12,6
92	4,47	13,7	226	2,31	1,34	0,77	114	37,64	3,5	664,0	74	7,11	4,53	5,0
93	2,47	11,4	96	1,37	0,61	1,08	71	40,72	3,4	371,0	82	7,06	3,76	18,1

94	5,01	16,2	263	3,14	1,48	0,92	110	9,88	4,2	959,2	98	6,35	3,91	1,1
**Values below reference**	15(16%)	30(32%) F/M=11/19	13(14%)	8(8,5%)	21(22,5%)	6(6,5%)	19(20%)	49(52%)	32(34%)	6(6,5%)	26(27,5%)	18(19%)	16(17%)	0(0%)
**Values above reference**	8(8,5%)	0(0%)	1(1%)	5(5,5%)	0(0%)	15(16%)	0(0%)	10(10,5%)	4(4%)	18(19%)	9(9,5%)	1(1%)	0(0%)	28(30%)

Journal of
Clinical Medicine

Table S3. Correlations of age of first symptoms, diagnosis and BMI

		diagnosis	age of first symptoms	BMI
Diagnosis	Pearson's r	—		
	p-value	—		
Age of first symptoms	Pearson's r	0.678	—	
	p-value	< .001	—	
BMI	Pearson's r	0.196	0.273	—
	p-value	0.060	0.008	—

BMI weakly but statistically significantly ($p<0.05$) correlates with the age of onset of symptoms.
